# Comprehensive Biochemical, Physiological, and Transcriptomic Analyses Provide Insights Into Floral Bud Dormancy in *Rhododendron delavayi* Franch

**DOI:** 10.3389/fgene.2022.856922

**Published:** 2022-05-17

**Authors:** Lu Zhang, Jie Song, Lvchun Peng, Weijia Xie, Shifeng Li, Jihua Wang

**Affiliations:** ^1^ Flower Research Institute of Yunnan Academy of Agricultural Sciences, Kunming, China; ^2^ National Engineering Research Center for Ornamental Horticulture, Kunming, China

**Keywords:** transcriptomics, dormancy, Rhododendron delavayi, physiological indices, phytohormones, WGCNA

## Abstract

Due to a scarcity of relevant data, the ornamental woody flower *Rhododendron delavayi* Franch. is examined in the current study for its low temperature-induced floral bud dormancy (late October–end December) aspect. This study used transcriptome data profiling and co-expression network analyses to identify the interplay between endogenous hormones and bud dormancy phases such as pre-dormancy, para-dormancy, endo-dormancy, eco-dormancy, and dormancy release. The biochemical and physiological assays revealed the significance of the abundance of phytohormones (abscisic acid, auxin, zeatin, and gibberellins), carbohydrate metabolism, oxidative species, and proteins (soluble proteins, proline, and malondialdehyde) in the regulatory mechanism of floral bud dormancy. The transcriptome sequencing generated 65,531 transcripts, out of which 504, 514, 307, and 240 expressed transcripts were mapped uniquely to pre-, para-, endo-, and eco-phases of dormancy, showing their roles in the stimulation of dormancy. The transcripts related to *LEA29*, *PGM*, *SAUR* family, *RPL9e*, *ATRX*, *FLOWERING LOCUS T*, *SERK1*, *ABFs*, *ASR2*, and *GID1* were identified as potential structural genes involved in floral bud dormancy. The transcription factors, including *Zinc fingers*, *CAD*, *MADS-box* family, *MYB*, and *MYC2,* revealed their potential regulatory roles concerning floral bud dormancy*.* The gene co-expression analysis highlighted essential hub genes involved in cold stress adaptations encoding proteins, *viz*, *SERPIN*, *HMA*, *PMEI*, *LEA_2*, *TRX*, *PSBT*, and *AMAT*. We exposed the connection among low temperature-induced dormancy in floral buds, differentially expressed genes, and hub genes *via* strict screening steps to escalate the confidence in selected genes as being truly putative in the pathways regulating bud dormancy mechanism. The identified candidate genes may prove worthy of further in-depth studies on molecular mechanisms involved in floral bud dormancy of *Rhododendron* species.

## Introduction

**FIGURE 1:**
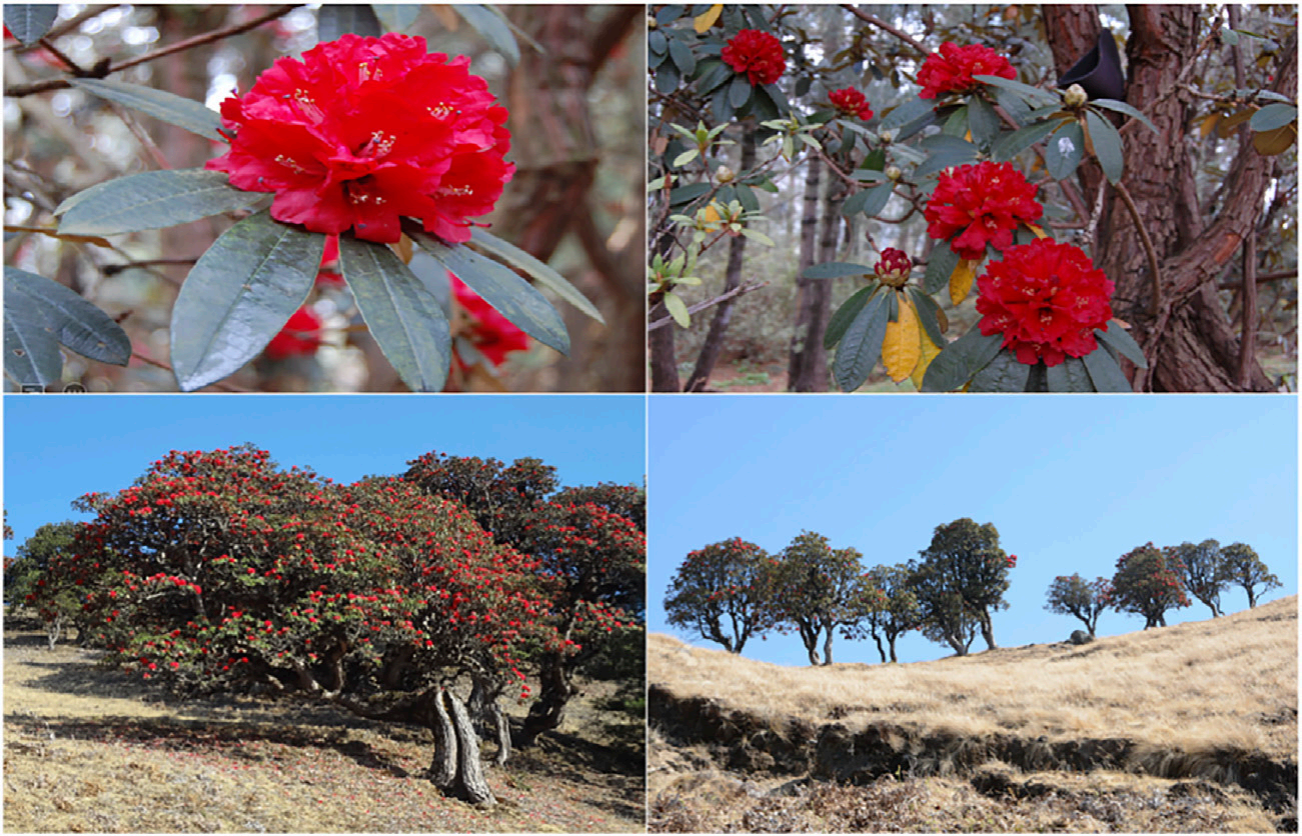
*Rhododendron delavayi* in its natural habitat in Cangshan, Dali, Yunnan, China.

*Rhododendron delavayi* Franch., of *Hymenanthes* subgenus, Ponticum section, an alpine woody ornamental plant from flowering species (spp.), belongs to the *Rhododendron* family, which includes the evergreen *Rhododendron* subgenus, the most diverse and most extensive genera in the family. Members of this family are found primarily in the Northern hemisphere, reaching the Asian tropics and the 1,200–3,200 m high the plateaus of Guizhou and Yunnan in Southwest China (Chamberlain et al., 1996; Fang et al., 2005). *R*. *delavayi* var. peramoenum and *R*. *delavayi* var. delavayi are two member varieties of this genus that have narrow leaves (former) prevalent in western Yunnan and comparatively broad leaves (later) flourishing widely in the rest parts of China (Fang et al., 2005; Sharma et al., 2014; Yang et al., 2020). They usually grow on sloppy rocks that have broad-leaved forests (Paul et al., 2018). The *R*. *delavayi* var. delavayi is widely spread to an altitude of 1200–3200 m due to more human activities; however, the wild relatives grow primarily near mountain peaks, along the cropland boundaries, or in the distant fragment forests (Zhang Y. et al., 2017). With this growing area base, *R*. *delavayi* var. delavayi is more prone to habitat fragmentation coupled with anthropogenic haphazardness (Figure 1).

The events in the lifecycle of plants dependent on the season and time are essential phenomena. The changes in the annual temperature of polar and temperate-climate regions greatly influence the development of reproductive structures in the flora (Fenner, 1998). The seasonal changes occurring throughout the year play a crucial role in making the plants adaptable to the ecological variations (Schwartz, 2003). Phenology is among the characteristics through which a plant or any organism responds to climatic changes (Miller-Rushing et al., 2010). The climatic factors that trigger phenological events in plants include temperature, precipitation, photoperiod, and soil (Blake and Harris, 1960; Paul et al., 2018). Similar to *R. delavayi* spp., for the onset of floral bud dormancy is cause by a decrease in temperature in their environment (Paul et al., 2011).

Though the sequence of events involved in pollen development remains conserved across flowering plants, the duration of developmental phases varies among them, ranging from a few weeks to a whole year (Vinckier et al., 2012). The cycle of development in almost all flowering plants undergoes a physiological phase of suspended growth at a particular stage or during establishing specific structures, *viz*, seeds, buds, or tubers, called “dormancy.” Regarding proper flowering, temperate perennials are characterized by a renowned phenomenon of chilling requirements followed by release from dormancy (Guo et al., 2014; Ferlito et al., 2021). Even today, limited knowledge is available on the mechanisms of plants involving temperature-sensitive gametophyte development, especially in temperate regions (Khodorova et al., 2010). Several studies were conducted on the flowering process of *Rhododendron* spp. (Christiaens, 2014; Lamsal and Welch, 2016; Wang et al., 2018), especially on cold tolerance (Panthi et al., 2021). The minimum temperature recorded for the onset of dormancy is <13°C, prevailing across 6 weeks (Barua, 1969; Vyas et al., 2007).

Most woody tree species undergo a bud (over-wintering) dormancy phase, a state of bud growth cessation, to endure adverse fluctuations in temperature and photoperiod (Salama et al., 2021). From a physiological perspective, this dormancy is categorized into para-dormancy, endo-dormancy, and eco-dormancy based on the signals originating from other plant structures (Lang et al., 1987) towards the flower bud for inhibition of growth; from the bud itself, and by environmental factors (low temperature prevent) (Rohde and Bhalerao, 2007). These signals are endogenous phytohormones such as abscisic acid (ABA), auxin (IAA), gibberellins (GAs), cytokinins and/or zeatin, *etc*. ABA is a renowned growth inhibitor phytohormone found in higher amounts within dormant structures. On the other hand, IAA, Gas, and cytokinins are dormancy-breaking phytohormones produced in the dormant buds or tubers for the re-initiation of sprouting (Hemberg, 1949; Skoog and Miller, 1957). Similarly, antioxidant signaling, including superoxide dismutase (SOD), catalases (CAT), and carbohydrates, as well as starch-cum-sugar contents, proteins, and proline concentrations inside dormant structures, plays crucial roles during and post-dormancy stages (Dimalla and Staden, 1977; Benkeblia et al., 2008; Gholizadeh et al., 2017). The molecular regulation of bud dormancy is variable and intricate (Zhanying et al., 2005; Cai et al., 2015; Liu and Sherif, 2019) its exact molecular mechanisms and essential genes need to be discovered (Suttle, 2007; Zhu et al., 2021).

Microarray hybridizations, cDNA, and ESTs sequencing libraries were constructed and utilized to deduce transcriptome profiles (mRNA + non-coding RNA) responsible for *Rhododendron* tissues and developmental stages involved in the dormancy phenomenon (Hartmann et al., 2011; Liu et al., 2012). The sequencing of transcriptome (mRNA) data *via* the high-throughput technique is undoubtedly swift and reliable (Wang et al., 2009; Macmanes, 2014; Pan et al., 2021). One possible worthwhile way to use mRNA-sequencing data is to identify interaction networks of co-expressed putative eigengenes/hub genes running the regulatory mechanisms of living systems biology *via* Weighted Gene Co-Expression Network Analysis (WGCNA). It is a valuable and convenient technique to identify potential candidate genes within the interactive networks of co-expressed genes/transcripts regulating specific mechanisms. In earlier studies, WGCNA has been utilized to identify key genes involved in photosynthesis and cell wall transport mechanisms (Zhou et al., 2020) and flowering development in *Rhododendron* spp. (Wang et al., 2018). The bud dormancy mechanism has been investigated in other plant species (Wang et al., 2020; Li P. et al., 2021) but not yet in *R*. *delavayi*. Hence, this study is novel in using this technique to determine the floral bud dormancy phenomenon.

In the context of abrupt fluctuations in climatic conditions, it is of high physiological and ecological significance to research the genes involved in the regulatory mechanism of winter dormancy in *Rhododendron*. This study used a high-throughput RNA-seq platform to generate transcriptomic information concerning the dormancy of floral buds at varying phases to profile the differentially expressed genes in dormant bud tissues. The purpose was to arrange mRNA-sequencing data during dormancy till sprouting to assist in annotating the *Rhododendron* genome for further research related to bud dormancy and flowering mechanism regulation. A series of computational tools working on principles of bioinformatics were operated for the purpose of assessing annotations and classification based on the functions of genes depicting differential expression related to R. delavayi floral bud dormancy. Consequently, the identified key genes may prove helpful in laying the foundation for in-depth studies to elaborate on the molecular mechanism of floral bud dormancy.

## Materials and Methods

### Plant Materials, Sample Collection, and Histology

The plant material utilized for the study was whole floral buds, collected from a 50-year-old tree of *Rhododendron delavayi* Franch. in the Kunming Golden Temple area under normal/natural conditions. The Temple is situated on the Mingfeng Mountain in Kunming city (102°43′ 5.99″E, 25°02′ 20.00″N) of Yunnan province, Southwest China. The samples were collected in three biological replicates from different branches of the tree at five stages of bud dormancy. The diameter and length of eight sampled floral buds were measured to check their growth rates of progression. The floral buds slowed down their growth starting form late August and entered into the phase of pre-dormancy at the end of September. Then they started to cease the growth process, entering the para-dormancy phase (Dormancy Initiation-DI: September 30th to October 29th), which continues till the start of December with a complete cessation in growth and development, so they achieved the endo-dormancy phase (dormancy maintenance-DM: October 30th to December 2nd). Then, a slow and steady increase in bud size and growth could be observed, as per temperature changes, falling into the eco-dormancy stage (dormancy release initiation-RI: December 3rd to December 27th), which completely diminished in mid-January, giving way to the release phase (full release from dormancy-FR: December 28th to January 14th). The sampled floral buds were immediately kept in liquid nitrogen after being wrapped in aluminum foil and then stored at −80°C for further analyses, including physiological and hormonal content determination and RNA extraction for transcriptome sequencing. The histological assays were conducted by fixing and embedding the sampled dormant bud tissues in wax. This was done to harden the tissues for the easy cutting of buds into different longitudinal and transverse sections. After staining of these tissues, they were examined under a light microscope to distinguish different tissue components.

### Physiological Indicators and Hormones

Estimating and quantifying hormones and physiological indices were carried out to mine for the regulatory mechanisms involved in the onset and release of the dormancy phenomenon. In this regard, four endogenous hormones, namely, ABA, zeatin, IAA, and GAs, were quantified from the sampled floral buds of different dormancy stages using the triple quadrupole LC-MS8040 platform following standard protocols (Xiao et al., 2020). Besides this, seven physiological indices were estimated, comprising soluble starch content (mg/g), soluble sugar content (%), soluble protein content (%), catalase (CAT) activity (U/g min), superoxide dismutase (SOD) activity (U/g.h), proline content (%), and malondialdehyde (MDA) content (mmol/g). The soluble sugar and soluble starch contents were determined by the anthrone colorimetry method (Dubois et al., 1956). The Coomassie Brilliant Blue (CBB) G-250 was utilized for the quantitation of soluble protein contents (Bradford, 1976). For the determination of SOD activity, nitro-blue tetrazolium (NBT) chemical was used *via* the light reduction method (Kono, 1978). CAT activity was estimated by using the Aebi protocol (Aebi, 1984). The Bates method (Bates et al., 1973) was used to determine the physiological status of plant entering dormancy by detecting the amount of free proline content. The MDA contents were estimated *via* the protocol mentioned earlier in studies (Wang et al., 2015; Zhang et al., 2021).

### cDNA Library Construction

Fifteen libraries were constructed from triplicated floral buds randomly selected at the five dormancy stages for transcriptome sequencing. The total RNA extraction was performed by following the protocol of TRIzol (TaKaRa, Dalian, China), and the extracted RNA was treated with DNase-I, Oligo (dT) to obtain mRNA. The quality assessment of extracted RNA regarding RNA integrity and contamination was estimated with the help of the Agilent 2100 Bioanalyzer system (Agilent Technologies, CA, United States) and 1% agarose gel electrophoresis, respectively. For the construction of paired-end libraries, 3 μg of RNA extracted from each sample was utilized and put in fragmentation buffer, followed by the preparation of cDNA from mRNA. The quantification and qualification of the sample library were assessed with the Agilent 2100 Bioanalyzer and the ABI StepOnePlus Real-Time System.

Moreover, according to the instructions recommended by the manufacturer (Illumina^®^), libraries were created by utilizing Kit. For RNA-Seq, the Illumina HiSeq technique was used. Furthermore, the paired-end libraries (read length: 150 bp) were sequenced using the Illumina HiSeq 2500 platform.

### Transcriptome Mapping

To attain clean reads, the reads containing short sequence length, i.e., ≤50 bp, adapters, poly-N, and low-quality (≥20% of bases with quality value ≤15) were eliminated *via* operating the FASTQ program (Cock et al., 2010). To expand the RNA-sequence data utility, contigs of triplicated samples from each dormancy phase were pooled together to assemble them to shape the non-redundant transcripts. The HISAT program v0.1.6-beta (Kim et al., 2015), a fast and sensitive spliced aligner, was utilized to align the transcripts parallel to the reference genome (Zhang L. et al., 2017).

### Novel Transcript Prediction

The transcript containing absent features in the reference annotation is termed as “novel transcript” (Zhang L. et al., 2017). It can be a new transcript with totally unknown features or a new isoform of a previously known gene. For the reconstruction of transcripts, StringTie v1. 0. 4 software (Pertea et al., 2015) was utilized, which was further compared against reference annotations using CuffCompare v2. 2. 1. For prediction related to coding potential hidden in the novel transcripts, merging these coding sequences with reference transcripts was accomplished to attain a complete and comprehensive reference. This practice was carried out with the help of CPC v0. 9-r^2^ software (Kong et al., 2007). The downstream analyses were based on this reference.

### Gene Expression Analysis and DEG Identification

The number of clean reads obtained after integrating reference transcripts with those of novel coding transcripts mapped onto the genome was estimated using Bowtie2 (v2. 2. 5) (Langmead and Salzberg, 2012). The number of fragments per kilobase of exon model per million mapped reads (FPKM) expected for each unigene was calculated based on the gene’s read count and gene length. The gene expression levels were calculated with the RSEM package (v1. 2. 12) (Li and Dewey, 2011) from RNA-Seq data. The differentially expressed genes (DEGs) between samples from different growth/dormancy stages were identified using the NOIseq package (Tarazona et al., 2011). The significant threshold *p*-value was estimated based on the false discovery rate (FDR) method to determine the reliability of gene expression. Observing the two-fold difference between FPKM values and FDR ≤0.001, the identified genes were taken as significant DEGs. The Pearson’s correlation among different samples was calculated using the R “cor” function.

### Pathway Analysis and TF Prediction

The functional annotation related to DEGs was attained *via* maximum BLAST hit against source databases, *viz*, Gene Ontology (GO), Kyoto Encyclopedia of Genes and Genomes (KEGG), and NCBI Non-redundant (nr) protein sequences. These pathway enrichment analyses were performed with the “Phyper” function of R. To extract the open reading frame (ORF) of each DEG, “get off v. EMBOSS 6. 5.7.0” software (Rice et al., 2000) was used. The obtained ORFs were then aligned against transcription factor domains with the help of “hmmsearch v3.0” (Mistry et al., 2013). The hierarchical clustering of DEGs was performed using R’s “pheatmap” function.

### Identification of Gene Networks

To identify particular modules harboring highly correlated genes, the WGCNA package of R was implemented (Langfelder and Horvath, 2008). The adjacency matrix was generated by using normalized FPKM values. The associations between gene modules and phenotypic data were calculated by utilizing the default settings of the WGCNA package and importing phenotypic data. Further, the Topological Overlap Matrix (TOM) was constructed using the conversion option within the WGCNA package from the already generated adjacency matrix. Next to the expression network building step was the classification of transcripts with more similar patterns regarding their expression into a single module, followed by calculating Eigen/hub/key genes for constructed modules. The calculated eigengenes of modules were exported out *via* Cytoscape v 3. 8. 2 export (Shannon et al., 2003).

### Validation *via* qRT-PCR

The RNA-seq data from Illumina sequencing was confirmed and validated by quantitative real-time PCR. The total RNA was taken out of floral bud tissues by following the total RNA Kit (BioTake, Beijing, China) manufacturer’s protocol, followed by its treatment with DNase-I. The cDNAs were synthesized from the reverse transcribed RNA with a cDNA Synthesis Kit (TaKaRa). The quantitative real-time PCR machine was used to accomplish the polymerase chain reaction (CFX96 Touch Real-Time PCR system, Biorad, Hercules, CA, United States). The qRT-PCR was accomplished by repeating each reaction three times and from three biological samples (Singh et al., 2021). The PCR was performed under the following conditions: 94°C (4 min) at the initial step, 94°C (15 s) for 40 cycles, 57°C (30 s), 70°C (30 s), 72°C (1 min) for the dissociation step, and a rise of 1°C at a regular interval of 5 s to shift from 75 to 90°C for acquiring the melting curve (Singh et al., 2020). Based on the gene annotation results, 19 candidate genes were selected from the identified DEGs and hub genes for quantitative RT-PCR analysis regarding their key roles in hormone signaling and regulatory pathways of floral growth, development, and bud dormancy (Supplementary Table S1). Specific primers were then designed and synthesized by Oligo v. 7. 0 software for the *Rhododendron* genes, and the elongation factor “S*l*EF” was utilized to perform as an internal control (Rotenberg et al., 2006). The specificity of primers was determined by a dissociative curve related to the individual gene. The 2^−ΔΔCt^ method was deployed to estimate the relative fold change of gene expressions (Livak and Schmittgen, 2001).

## Results

### Assessment of Dormancy Phases

**FIGURE 2:**
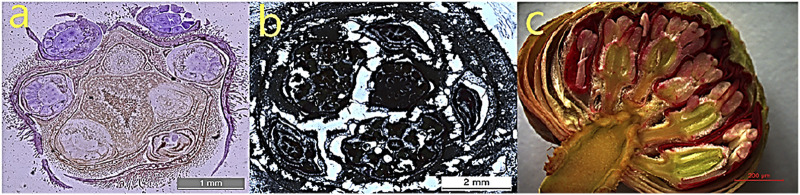
Histological assay through light microscopy showing *Rhododendron delavayi* Franch. dormant floral bud initiation in paraffin section and freehand longitudinal section. **(A)** Cross-section (1 mm) of buds collected on *1*) 1st September, and *2*) 29th October, containing the loose tissue structure due to the large-sized floral bud, making it challenging to take the paraffin section **(B)** part of the image (2 mm) showing spread out of stain. **(C)** The longitudinal section (200 µM) of the sampled bud depicts the florets at the stage of complete differentiation of flower buds, which then gradually enters the dormancy phase.

Based on bud morphology, five different growth cessation phases were identified, i.e., pre-dormancy, para-dormancy, endo-dormancy, eco-dormancy, and release from dormancy. The observed differences in floral buds’ size and growth rate before, during, and after the dormancy period were diagrammatically and graphically illustrated. The pictorial summary exhibited paraffin and freehand longitudinal sections of floral buds from September to the end of October. The diagrams showed that the floral buds had completed the differentiation among the inflorescence parts before entering the dormancy phase (Figure 2).

**FIGURE 3:**
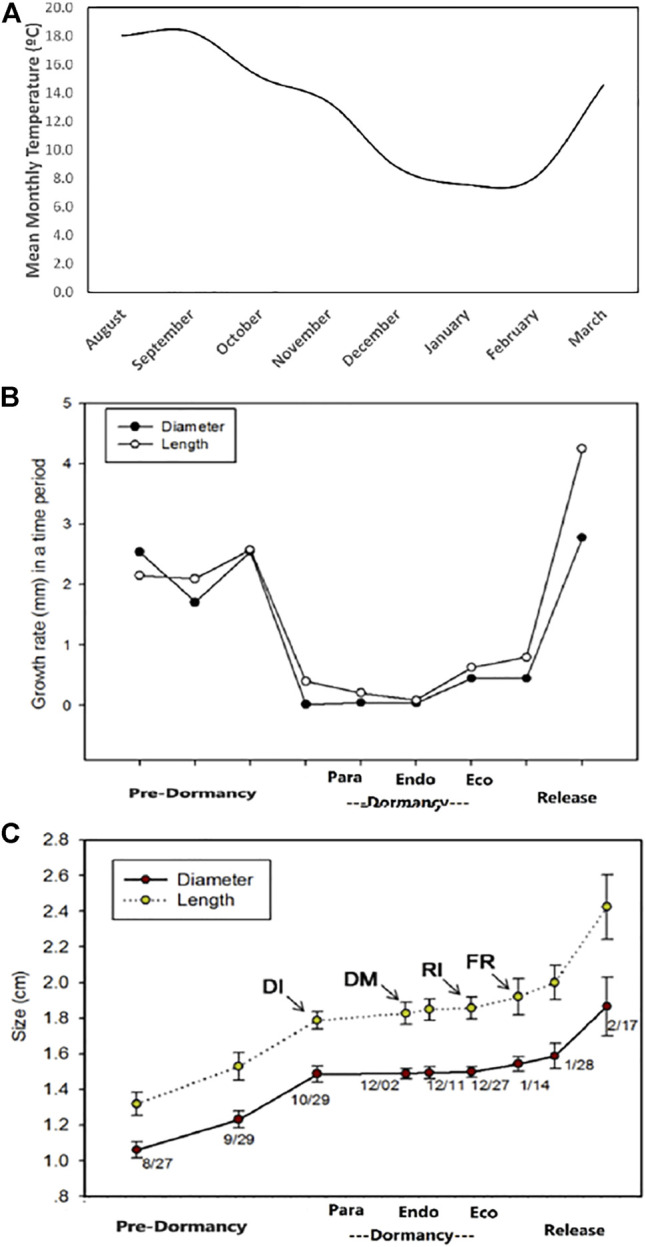
The detailed fluctuating average temperature (°C) ranges before, during and at the end of dormancy phases on **(A)** monthly average **(B)** the different growth rates during dormancy phases: pre-dormancy: flower buds growth becomes slow before the initiation of dormancy; dormancy initiation (DI) or para-dormancy: floral bud ready to enter deep dormancy phase; dormancy maintenance (DM) or endo-dormancy phase: floral bud during the deep sleep; dormancy release initiation (RI) or eco-dormancy; flowering release (FR) or dormancy release period **(C)** changes in the floral bud sizes during different dormancy phases.

The meteorological details, including average temperature (°C), ranged daily and fortnightly during the experimental period, were presented in Figures 3A,B. The graph depicted the end of September as an initiation point for the size and growth rate decline. The cessation in growth started at the end of October, marked as dormancy initiation (DI). The dormancy maintenance (DM) phase continued till the start of December; then, a change in the size of the floral bud was observed from mid-to-end of December, shifting the bud growth rate towards the dormancy release initiation-RI-Phase. Finally, dormancy broke during the mid-to-end of January, bringing back the floral bud in its normal growth period (Figures 3C,D).

### Changes in Physiological Indicators and Hormones

**FIGURE 4:**
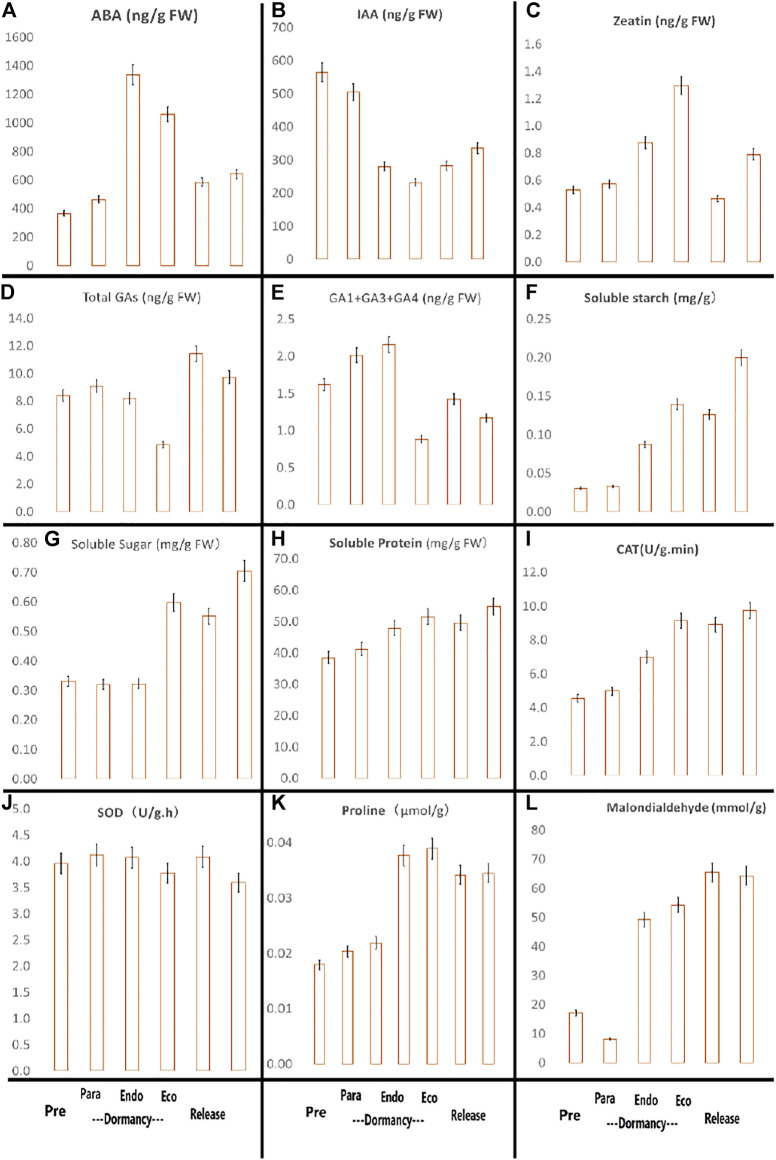
Contents of endogenous phytohormones **(A)** ABA (ng/g FW: fresh weight), **(B)** IAA (ng/g), **(C)** zeatin (ng/g), **(D)** total GAs (ng/g), **(E)** bioactive GAs (GA1+GA3+GA4) **(F)** soluble starch (mg/g), **(G)** soluble sugar (mg/g), **(H)** soluble protein (mg/g), **(I)** CAT activity (U/g.min), **(J)** SOD activity (U/g.h), **(K)** proline contents (µmol/g), **(L)** MDA content (mmol/g) in flower buds during different dormancy phases.

We estimated the content of four types of endogenous hormones, *viz*, ABA, IAA, cytokinins and/or zeatin, Gas, *etc*., in floral bud tissues during the mentioned bud dormancy phases. Moreover, seven physiological indices, i.e., soluble protein content, soluble starch content, soluble sugar content, CAT, SOD, proline content, and MDA, were also quantified during the bud dormancy phases. The ABA and zeatin hormones increased during dormancy phases, while IAA and GAs content lowered during dormancy phases from October to December (Figure 4). Similarly, an increase in soluble starch, soluble sugar content, soluble protein content, CAT, proline content, and MDA content was observed. However, SOD decreased during this period of dormancy (Figure 4).

### Transcriptome Profiling

The bud samples were collected in triplicate from the five dormancy stages. The number of raw reads obtained after RNA sequencing was about 51.55 million reads, filtered *via* removal of adaptors and ambiguous or low-quality reads. Consequently, approximately 49 million (95%) clean reads were obtained (Supplementary Table S2). On average, 4.43 Gb of clean data was obtained from each bud sample, with a Q20% greater than 99.07% and a Q30% greater than 96.85% (Supplementary Table S2). The clean base data contained GC content ranging between 46.8 and 48.37% (Supplementary Table S3). After assembling, almost 82.8% of the clean reads were aligned against the reference genome (Zhang L. et al., 2017), using the HISAT program (Kim et al., 2015). The analysis provided 65,531 transcripts, and 39,166 novel transcripts were detected (Supplementary Table S4).

**FIGURE 5:**
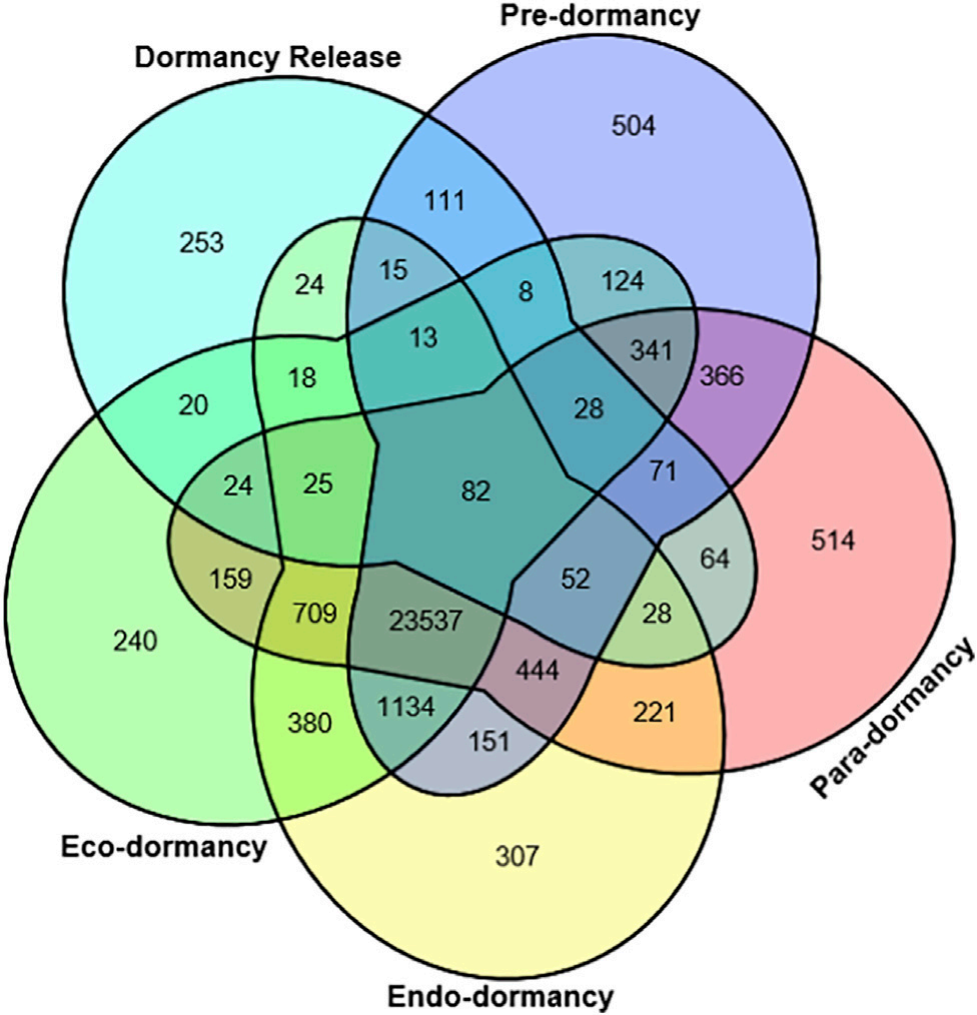
Venn diagram representing common and unique transcripts among multiple dormancy stages, *viz*, pre-dormancy, para-dormancy, eco-dormancy, endo-dormancy, and dormancy release.

The novel transcripts were then merged with reference transcripts to attain a complete reference from which the clean reads were mapped to calculate the expression of genes. The Venn diagram depicts the expressed transcripts identified uniquely at each dormancy phase (504, 514, 307, 240, and 253 uniquely expressed transcripts at pre-dormancy, para-dormancy, endo-dormancy, eco-dormancy, and dormancy release phases, respectively). Except for the dormancy release , a total of 23,537 expressed transcripts were found common at all the four dormancy phases (Figure 5). Before moving towards comparative transcriptome analyses, the data of each transcriptome was evaluated regarding quantitation and quality control. A correlation analysis was conducted for this purpose. Almost all the bud samples at different dormancy phases revealed a highly significant correlation among themselves (Supplementary Figure S1). Such quality of results paved the way further towards transcriptome profiling of genes related to the regulation of floral bud dormancy.

### Detection of Associated DEGs

**FIGURE 6:**
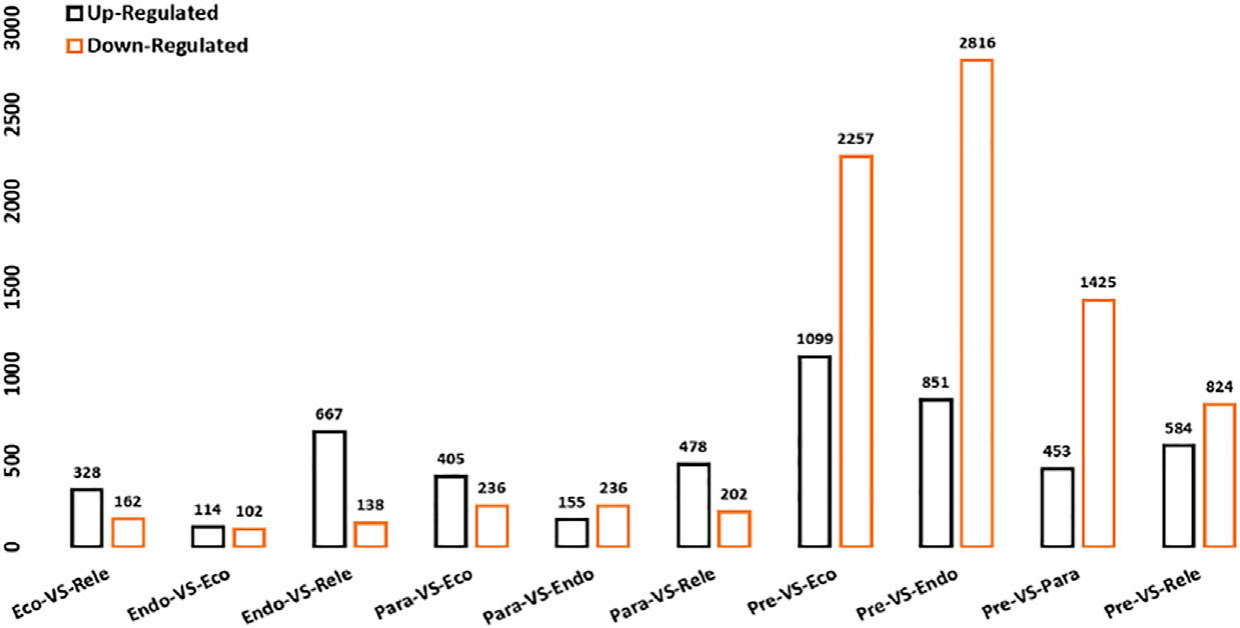
Summary of Differentially Expressed Genes (DEGs); *X*-axis displayed differential expression patterns in all possible pairwise comparisons of bud dormancy samples. The *Y*-axis represented the number of DEGs. Orange-colored: up-regulated DEGs; black color: down-regulated DEGs.

The gene expression profiles of sampled buds for growth and dormancy were estimated by the DEGseq algorithm to discover the differentially expressed genes (DEGs). A total of 13,542 DEGs were detected based on all possible pairwise comparisons of bud dormancy phases (Supplementary Table S5). Details of identified DEGs associated with following sets of comparison were, *viz*, pre-vs-para: 1878 (up: 24%, down: 76%), pre-vs-endo: 3667 (up: 23%, down: 77%), pre-vs-eco: 3356 (up: 33%, down: 67%), pre-vs-rele: 1408 (up: 41%, down: 59%), para-vs-endo: 391 (up: 40%, down: 60%), para-vs-eco: 641 (up: 63%, down: 37%), para-vs-rele: 680 (up: 70%, down: 30%), endo-vs-eco: 216 (up: 53%, down: 47%), endo-vs-rele: 805 (up: 83%, down: 17%):, and eco-vs-rele: 490 (up: 67%, down: 33%) (Figure 6 and Supplementary Table S6).

### Annotation and Investigation of DEGs

Comparative transcriptomics identified 405 DEGs conserved between different comparison groups of floral bud dormancy phases (Supplementary Table S6). The conserved genes were selected based on the expression conserved between at least two comparisons. Among the 405 genes, 4, 4, 29, 30, 14, 21, 2, and 300 were identified for floral bud dormancy, flowering initiation, IAA biosynthesis, embryogenesis, ABA biosynthesis, GA synthesis, zeatin, cytokinesis, and transcription regulation, respectively (Supplementary Table S7). The four floral bud dormancy transcript genes were *PORCN*, *LEA29*, *TRMT2A*, and *ATRX*.

We further explored the conserved genes based on KEGG annotation and identified four genes encoding *FLOWERING LOCUS T* (*FLT*) and *elongation factor 1 alpha-like protein*. All four genes related to floral initiation were down-regulated during all dormancy phases. A total of 29 genes were associated with auxins. Most genes like the *SAUR* family, *RPL9e*, and *GH3* were related to IAA, depicting their role in sustaining the dormancy period (Supplementary Table S8). Similarly, comparative transcriptomics suggested genes related to embryogenesis, *viz*, *GST*, *ATRX*, *SLC39A1*, *SERK1*, *PGM*, *LEA29*. They were down-regulated during all floral bud dormancy stages while up-regulated in the release phase, suggesting their role in the flower developmental process except for *PGM* and *LEA29,* which were up-regulated in early dormancy phases, inhibiting the floral development process. The KEGG annotation corresponding to these genes emphasized their roles in embryogenesis during dormancy and dormancy release. The remaining 20 genes were down-regulated during the initial dormancy stages, including para-dormancy, endo-dormancy, and eco-dormancy (Supplementary Table S8).

We further studied the genes associated with ABA synthesis, GA synthesis, cytokinesis, and transcription regulation. Embryonic ABA synthesis played a critical role during dormancy. The identified DEGs concerning ABA synthesis, i.e., *ASR2*, *ABFs,* which were down-regulated during the deep dormancy period, including endo-dormancy, para-dormancy, and eco-dormancy, induced the high ABA production to promote dormancy. They were up-regulated at the release of dormancy to lower the production of ABA for the breaking of dormancy. However, the transcripts of the *SAUR* family and *RPL9e* showed up-regulated expression patterns during dormancy. Late embryogenesis abundant (*LEA*) protein was identified with an up-regulated expression pattern throughout the dormancy period. Few gibberellin-induced proteins (related to GA synthesis) such as GID1 were identified with down-regulated expression patterns during the deep dormancy period but up-regulated at the dormancy release phase (Supplementary Table S7).

Among 301 transcription factors (TF), the most prominent TFs of identified DEGs were zinc finger (38), *CAD* (26), *MADS*-*box* (24), *MYB* (17), *WRKY* (9), *HSP* (7), *MYC2* (6), and *SPT5* (6). Most TFs depicted a trend of down-regulation during dormancy while up-regulated at the dormancy release phase. However, temperature-sensitive TFs were up-regulated during the dormancy period but did not express themselves at later stages of dormancy release (Supplementary Table S7).

### Identification of Connected Hubs and Gene Modules

**FIGURE 7:**
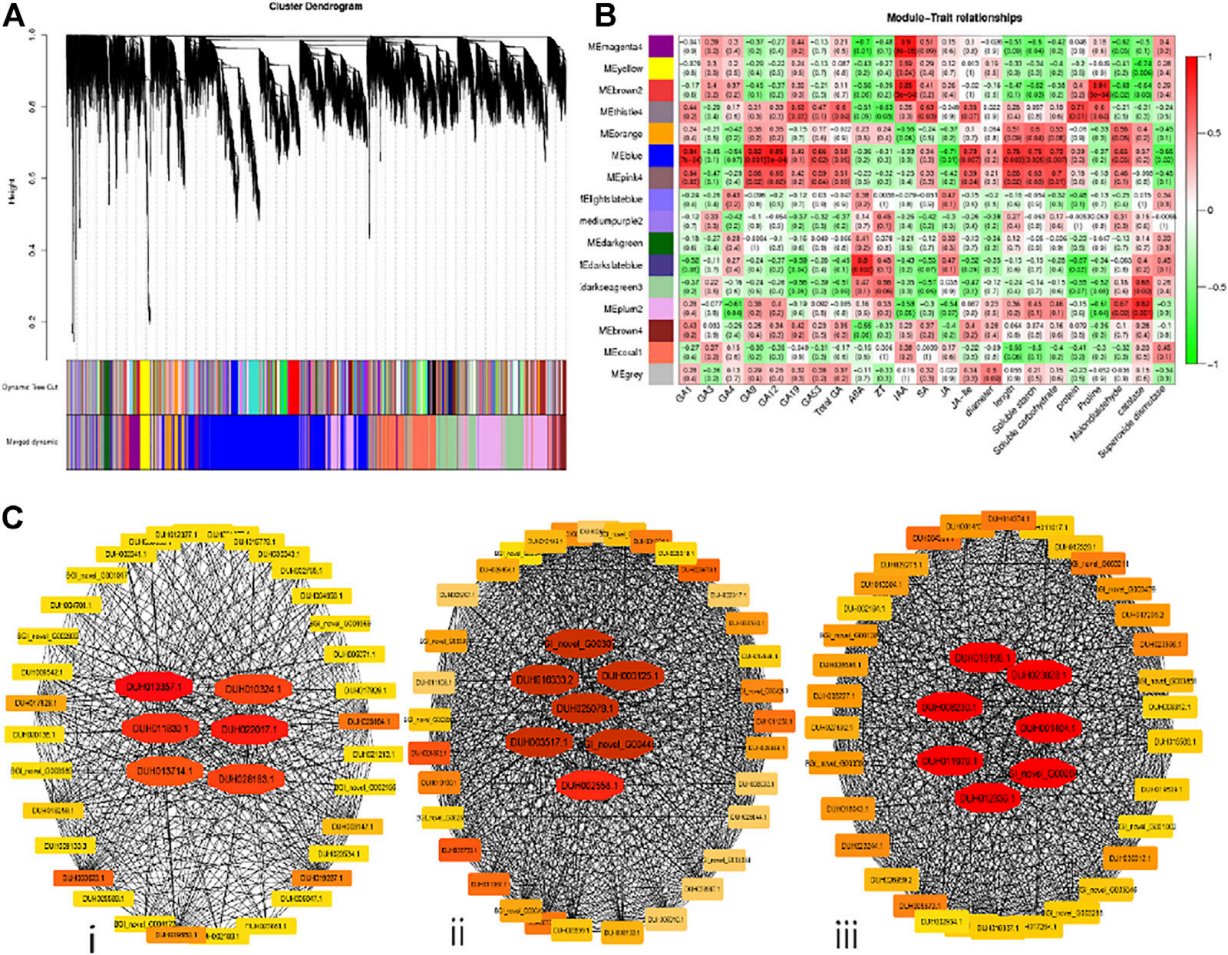
WGCNA has driven eigengenes-based gene networks related to the regulation of floral bud dormancy in *R*. *delavayi* Franch. **(A)** Clustering *via* a hierarchical approach reveals 16 resulting modules harboring co-expressed weighted genes. Each gene in the modules corresponds to a separate individual leaflet of the cluster tree. **(B)** Association chart between traits and modules utilizing Pearson correlation coefficient ranging between −1 to 1with green to red gradient. **(C)** Interaction network for the three eigengenes/hub genes modules related to phenotypic, morphological, and biochemical traits scored to get information related to the regulation of bud dormancy in *R*. *delavayi*. The candidates’ eigengenes/hub genes localized in the depicted networks for **(i)** blue, **(ii)** pink4 and **(iii)** thistle4 modules are tinted with red due to their higher weights than other genes in the orange to yellow gradient.

The weighted gene co-expression network analysis (WGCNA) is the analytical tool to define correlation networks among variables for connecting them and clustering the highly co-expressed ones for shaping modules. The FPKM values of expressed genes related to the four stages were utilized in WGCNA to expose highly correlated genes. An adjacency matrix was constructed based on DEGs, and 16 discrete modules of genes related to phenotypic, physiological, and biochemical traits expressed for the regulation of floral bud dormancy were generated. Each module represented a cluster of genes depicted with arbitrary colors in the form of a cluster gram coupled with a network heatmap (Figure 7).

Out of the 16 modules, six significantly correlated with phenotypic, physiological, and biochemical traits. It suggests a potential role in the regulation of floral bud dormancy. The MEblue, MEpink4 and MEthistle4 modules displayed the maximum positive correlations with the traits investigated. The details of transcript-based modules were graphically elaborated in Figure 7. Selection of genes from these three modules was accompanied by estimation of their nodes and edges with the help of the R package WGCNA to visualize gene networks. Visualization of gene networks was performed by exporting the generated information into Cytoscape. We further moved towards recruiting the top 40 putative genes from the three significantly correlated gene modules, thus contributing to primary functions in the gene networks (Figure 7). The MEblue module comprised six top eigengenes (hub genes), *viz*, *DUH013357.1*, *DUH022017.1*, *DUH011830.1*, *DUH010324.1*, *DUH013714.1,* and *DUH028183.1*. The MEpink4 module revealed seven hub genes, i.e., *BGI_novel_G003073*, *DUH016333.2*, *DUH003125.1*, *DUH025079.1*, *DUH003517.1*, *DUH002558.1,* and *BGI_novel_G004463*. The METhistle4 module also contained seven hub genes, i.e., *DUH019196.1*, *DUH008230.1*, *DUH0238828.1*, *DUH001404.1*, *DUH011979.1*, *DUH012339.1* and *BGI_novel_G002949* (Figure 7). A hub gene from the blue module *DUH022017*.1 encoded Glycine-rich RNA binding protein (GR-RBP), which was produced as a cold shock response. *DUH011830*.*1* encoded heavy metal-transporting ATPases (HMAs) involved in flower development by absorbing minerals or heavy metals from intracellular membrane-bound organelles. *DUH010324.1* hub gene from the blue module encodes pectin methylesterase inhibitor (PMEI), with functions in the metabolism of sucrose, starch, and carbohydrate catabolism by regulating the activity of enzymes.

A hub gene from the pink4 module, *viz*, *DUH003125*.*1,* encodes a late embryogenesis abundant (LEA_2) protein with a function in transport and catabolism of protein from other plant parts to floral buds to regulate the dormancy phenomenon. *DUH016333.2* and *DUH003517.1* from pink4 module encodes a SERPIN protein and transmembrane protein, playing functions of RNA transport in defense mechanisms and metabolism of terpenoids and polyketides. *DUH011979.1* hub gene from the thistle4 module encoded Thioredoxin (TRX) protein with oxidoreductase activity during the floral bud dormancy period. Another hub gene, *DUH023828.1* from the thistle4 module, encodes Photosystem II 5 kDa protein (PSBT) with a significant role in dark reactions of photosynthesis, but does not use sunlight energy directly from the sun. One hub gene from the thistle4 module, *DUH008230.1,* encoded Methanol O-anthraniloyltransferase (AMAT), has a function of linolenic acid metabolism and related transferase activity. There are five hub genes, i.e., *BGI_novel_G004463*, *DUH019196*.*1, DUH002558.1*, *BGI_novel_G003073*, and *DUH001404.1*, with either uncharacterized proteins or with unknown functions.

### Candidate Gene Validation *via* qRT-PCR

**FIGURE 8:**
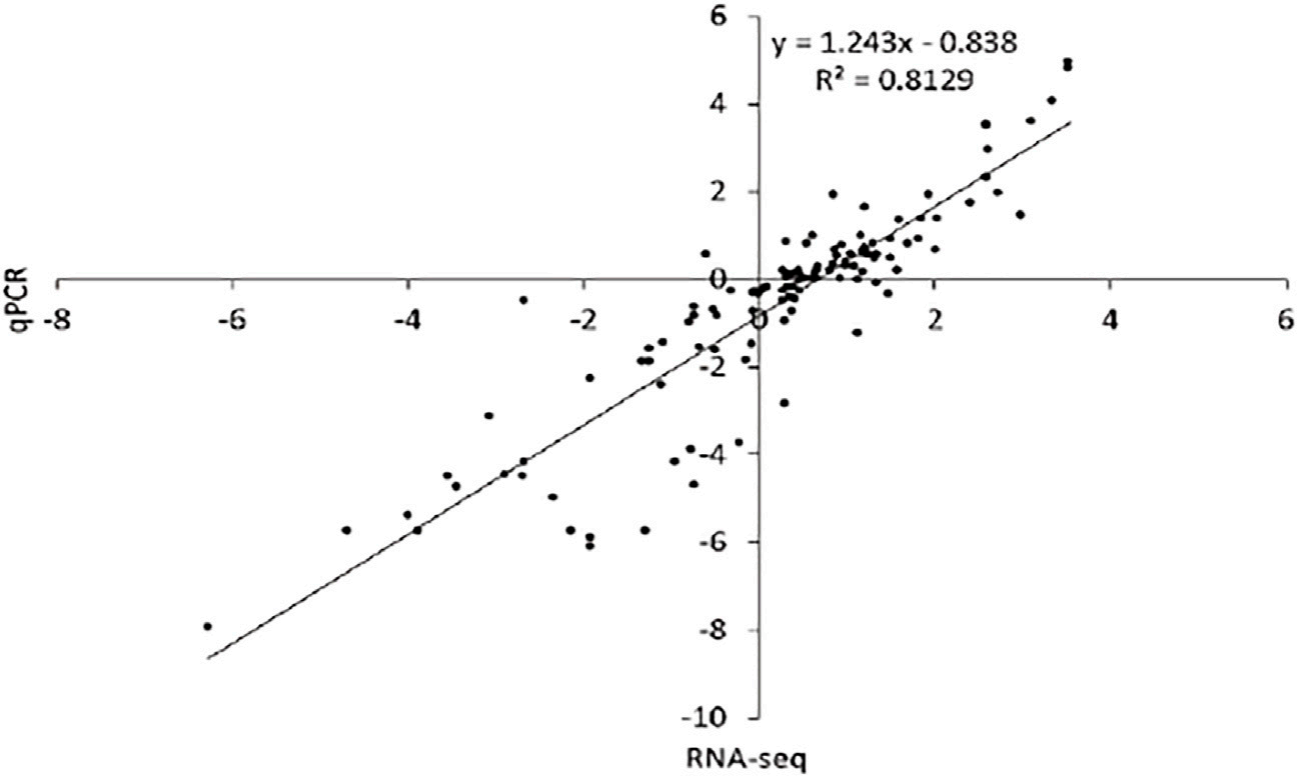
Validation of expression results using qRT-PCR represents the correlation between qRT-PCR and RNA-seq expression results for randomly selected DEGs related to five dormancy stages considered under study.

The accuracy of the transcriptome sequencing data is the prerequisite for identifying differentially expressed genes and subsequent enrichment analysis of GO and KEGG functions. For transcriptome data validation, 19 genes with diverse expression patterns at the five floral bud dormancy stages were chosen for the quantitative PCR analysis. qRT-PCR analysis revealed that the trend of gene expression by transcription measured (FPKM) and relative expression by the qRT-PCR method was consistent, and a commendable correlation, i.e., *R* ^2^ = 0.8129, has been detected between the pair of qRT-PCR data and RNA-seq data (Figure 8). This amount of correlation suggests the reliability of RNA-seq data gathered in the current bundle of studies.

## Discussion

Though the domestication and cultivation of plants have been given importance regarding food production, their ornamental aspect has not been neglected (Weigel et al., 1992; Noman et al., 2017). To improve the ornamental attributes of the *Rhododendron* genus, understanding genetic mechanisms and proper characterization of the flowering phenomenon is a significant step to be considered (Noman et al., 2017; Xiao et al., 2018). The current study revolved around the transcriptome data of physiological and biochemical pathways concerning floral bud dormancy phases to better understand their interplay. The flowering mechanism in plants is a multifaceted developmental process influenced by environmental and endogenous factors including, light, temperature, physiology, and phytohormones (Bodson, 1983; Vasconcelos et al., 2009). Considering the previous studies on *R*. *delavayi*, four stages of bud dormancy were examined by taking samples for transcriptomic analysis like earlier studies (Liu et al., 2015; Wang et al., 2018). Here, we assessed some of the biochemical and physiological phytohormones in *R*. *delavayi* to examine their interplay with the phenology of the dormancy mechanism. Our outcomes were concurrent with previous findings regarding the relative abundance of phytohormones, proteins and sugar metabolism, and antioxidant signaling in regulating the bud dormancy and sprouting phenomena.

The significant interplay of phenology and adaptation is controlled by regulatory phytohormones involving several redox signaling pathways (Considine and Foyer, 2014). The relative proportion of endogenous phytohormones like ABA, GAs, zeatins, IAA, starch, sugars, and proteins influences the regulation of the bud dormancy mechanism in several plants. The endogenous ABA, a growth inhibitor, was observed in higher concentrations within the dormant floral buds (Karssen et al., 1990; Claassens and Vreugdenhil, 2000). On the contrary, concentrations of endogenous IAA, zeatin and GAs were lower in the para- and endo-dormancy phases and increased at the dormancy release phase, acting as the dormancy breaker phytohormones (Lippert et al., 1958; Turnbull and Hanke, 1985; Sukhova et al., 1993). Likewise, the trend of increase or decrease in concentrations of biochemical compounds was consistent with earlier reports as per the natural phenomenon of bud dormancy. The amounts of soluble starch and sugar content (as energy sources) were observed in higher amounts during deep dormancy phases, which decreased at release or breakage of dormancy. The possible reasons behind might include the reduced activity of amylase for breaking starch/sugars in dormant buds, consequently giving a higher accumulation of starch/sugars, which then increases at the termination of dormancy (Davies, 1990; De Moraes et al., 2016; Gholizadeh et al., 2017). Redox signaling and protein contents were observed with increased concentrations such as SOD, CAT and soluble protein contents at the dormancy termination/release stage, proving their pivotal role as dormancy-breaking compounds (Pérez et al., 2008; Gholizadeh et al., 2017; Beauvieux et al., 2018; Mujahid et al., 2020). The amounts of CAT and MDA remained stable (steady increase) during endo-dormancy but the amino acid proline and went up regarding accumulation in bud tissues, protecting the cell against oxidative stresses and leading to pentose phosphate pathway inducing the release of dormancy phase (Tan et al., 2015; Mujahid et al., 2020).

A total of 23,537 transcripts were simultaneously associated with all the five bud dormancy phases, revealing their role regarding the induction of floral bud dormancy. Besides, 504, 514, 307, 240, and 253 transcripts were mapped uniquely to pre, para, endo, eco, and release phases, showing their roles in the induction or release of each respective dormancy stage. Such transcriptome studies on the exploration of regulatory mechanisms for floral organ development in plants have been published previously (Zhang Q. et al., 2017; Lucero et al., 2017; Noman et al., 2017; Ding et al., 2019; Duan et al., 2019; Liu and Sherif, 2019; Liu et al., 2020; Zalko et al., 2020; Zhou and Zhu, 2020; Liu et al., 2021; Zhu et al., 2021). Thorough investigations revealed putative variations in the expression levels of DEGs, representing their important roles in bud dormancy induction in response to low temperatures around them. The DEGs, i.e., *DUH023687.1*, encoded *PORCN*, *DUH011466.5* encoded *LEA29*, and *DUH023802* encoded transcriptional regulator *ATRX*. They were observed in the regulation of the bud dormancy phenomenon. *ATRX* was down-regulated during the critical dormancy phases, *viz*, para and endo, while up-regulated during developmental stages, suggesting its role in bud growth and development processes. This gene is also a transcriptional regulator in Arabidopsis and other flowering shrubs (Wang et al., 2018; Qian et al., 2020). The down-regulated genes (*FT* and *elongation factor 1 alpha-like protein*) during dormancy stages suggest their leading roles in flowering development and sensitivity to low temperatures (Abe et al., 2015; Li Z. et al., 2021).

As previously explained, IAA synthesis was significantly decreased during the dormancy period, with a slight increase at the dormancy release stage. This study reported almost 29 genes involved in the synthesis of *IAA* with a down-regulation pattern, suggesting their role in the continuation of the dormancy period. Auxin is essential for the growth and development of bud or apices (Carraro et al., 2006; Hartmann et al., 2011), but when down-regulated, these genes induced cessation of floral bud growth (Wang et al., 2018). Similar down-regulation trend in the IAA-related *SAUR f*amily, *RPL9e,* and *GH3* was observed to promote dormancy (Liu et al., 2015).

Comparative transcriptome profiles provided embryogenesis related key genes: *GST*, *ATRX*, *SLC39A1*, *SERK1*, *PGM*, and *LEA29*. They got down-regulated during all the dormancy phases except *PGM* and *LEA29* (Liu et al., 2015), highlighting their roles in the induction and maintaining deep sleep periods in response to low temperature (Hong-Bo et al., 2005; Pawłowski, 2007). The part usually played by phytohormones, *viz*, ABA, cytokinins, ethylene, and GAs, in thermo-sensitivity has been reported in earlier studies (Liu et al., 2015; Hui et al., 2017; Qian et al., 2020). In the current study, the ABA biosynthesis up-regulation was taken as an important outcome as it is a phytohormone with a significant role in seed, apical or floral bud dormancy (Meijón et al., 2009; Meijón et al., 2011) in response to temperature change in the surrounding environment (Chen et al., 2020). A total of four genes were found with up-regulation during the para, endo, and eco-phases. The opposite of ABA is GA, i.e., its high production its the growth. So it is observed here in the study that some DEGs showed down-regulation and consequently produced fewer GAs during the dormancy phase (Wang et al., 2018).

In recent decades, the captivating roles of TFs in the developmental process have been widely explored and elaborated (Riechmann and Ratcliffe, 2000; Dubos et al., 2010; Rushton et al., 2010). *MADS-box* gene family have a crucial role in the flowering development process (Kater et al., 2006), and they are widely scattered in the genome of flowering plants (Theissen et al., 2000). Its lower expression pattern implied their role of inhibiting floral development. Previously, the *MADS-box* gene family was also extracted from other species of *Rhododendron* (Cheon et al., 2011; Tasaki et al., 2019). Numerous genes involved in the flowering development process are produced in higher amounts at the bud stage. The prominent ones, *viz*, zinc finger, *MYB* (Zhou and Zhu, 2020), *WRKY* (Zhou and Zhu, 2020), *HSP*, *MYC2*, and *SPT5* showed down-regulation during deep dormancy phases. It infers their crucial roles for blooming thus, need a bit rise in temperature of the surrounding environment for their up-regulation to re-progress flower development (Wang et al., 2018).

Several hub genes identified in this study through WGCNA encode genes that synthesize specific secondary metabolites and play signal transduction activity to make the plant adaptable to the environmental fluctuations and ultimately in the regulation of the plant’s circadian rhythm. A hub gene from the blue module *DUH022017*.1 encodes *GR-RBP* produced as cold shock response. These proteins were reported earlier in abiotic stresses response, especially cold, to regulate circadian clock, resulting in delayed flowering and reproductive development, particularly by affecting FLC, a repressor of *MADS-*box observed in Arabidopsis, barley, rice, cauliflower, tobacco, carrot, (Steffen et al., 2019; Alptekin et al., 2021; Ma et al., 2021). Another hub gene *DUH011830*.*1* is responsible for the production of *HMA* proteins. These are the heavy metal-binding domain of “metallochaperones” produced in vascular plants, safely transporting metallic ions to specific sites in cell. These proteins are produced in response to environmental changes, including cold stress. The reported studies in Arabidopsis and Rice comprised their expressions in inflorescence and reproductive parts during abiotic stress conditions (De Abreu-Neto et al., 2013). *DUH010324*.1 hub gene encodes *PMEI* proteins, with significant roles in floral organ development. These *PMEIs* were reported previously in cabbage, pepper, Arabidopsis, and Brassica during abiotic stress conditions caused by environmental changes, including cold stress (Tan et al., 2018; Wormit and Usadel, 2018) by inhibition of the degradation of pectin in the cell wall integrity. *DUH003125*.*1* hub gene responsible for producing *LEA*_2 protein for transport of other proteins to floral parts during dormancy has earlier been reported in almond during bud dormancy (Prudencio et al., 2018) and Arabidopsis under environmental fluctuations (Hundertmark and Hincha, 2008). *DUH011979.1* hub gene coding for *TRX* proteins during the dormant period was found earlier in Arabidopsis under cold stress conditions (Calderón et al., 2018; Chibani et al., 2021).

## Conclusion

The current knowledge elaborated on the phenology and regulation of floral bud dormancy in *Rhododendron delavayi* Franch. based on physiological and biochemical endogenous hormones. Thorough investigations into the transcriptional profile of bud dormancy-related DEGs led to the identification of some key genes encoding *Zinc finger*, *MYB*, *WRKY*, *MYC2*, *SPT5*, *MADS-box*, *ATRX*, *FLOWERING LOCUS T*, and *elongation factor 1 alpha-like protein* transcripts. The co-expression patterns were evaluated, and some hub genes were discovered related to environmental fluctuation adaptations encoding proteins, *viz*, *SERPIN*, *HMA*, *PMEI*, *LEA*_2, *TRX*, transmembrane proteins, *PSBT*, and *AMAT* involved in flower development, carbohydrates, starch and fatty acid metabolism, transport, photosynthesis, and oxidoreductase activities. These genes are predicted to be involved in maintaining and regulating the floral bud dormancy phenomenon under cold stress conditions. Thus, the connection between these DEGs, hub genes, and low temperature-induced dormancy in floral buds was explored. These findings can pave the way towards a better understanding of the molecular mechanisms of floral bud dormancy in *Rhododendron.* The identified genes may prove a beneficial genetic resource regarding the evolution of floral traits in *Rhododendron* species, as there is no earlier evidence of their roles in controlling the bud dormancy mechanism in *Rhododendron*.

## Data Availability

The datasets presented in this study can be found in online repositories. The names of the repository/repositories and accession number(s) can be found in the article/Supplementary Material.
