# Supplementary figures and images for: Comprehensive Biochemical, Physiological, and Transcriptomic Analyses Provide Insights Into Floral Bud Dormancy in Rhododendron delavayi Franch
Source: Front Genet. 2022 May 17;13:856922. doi: 10.3389/fgene.2022.856922 (PMC9152171; doi:10.3389/fgene.2022.856922)

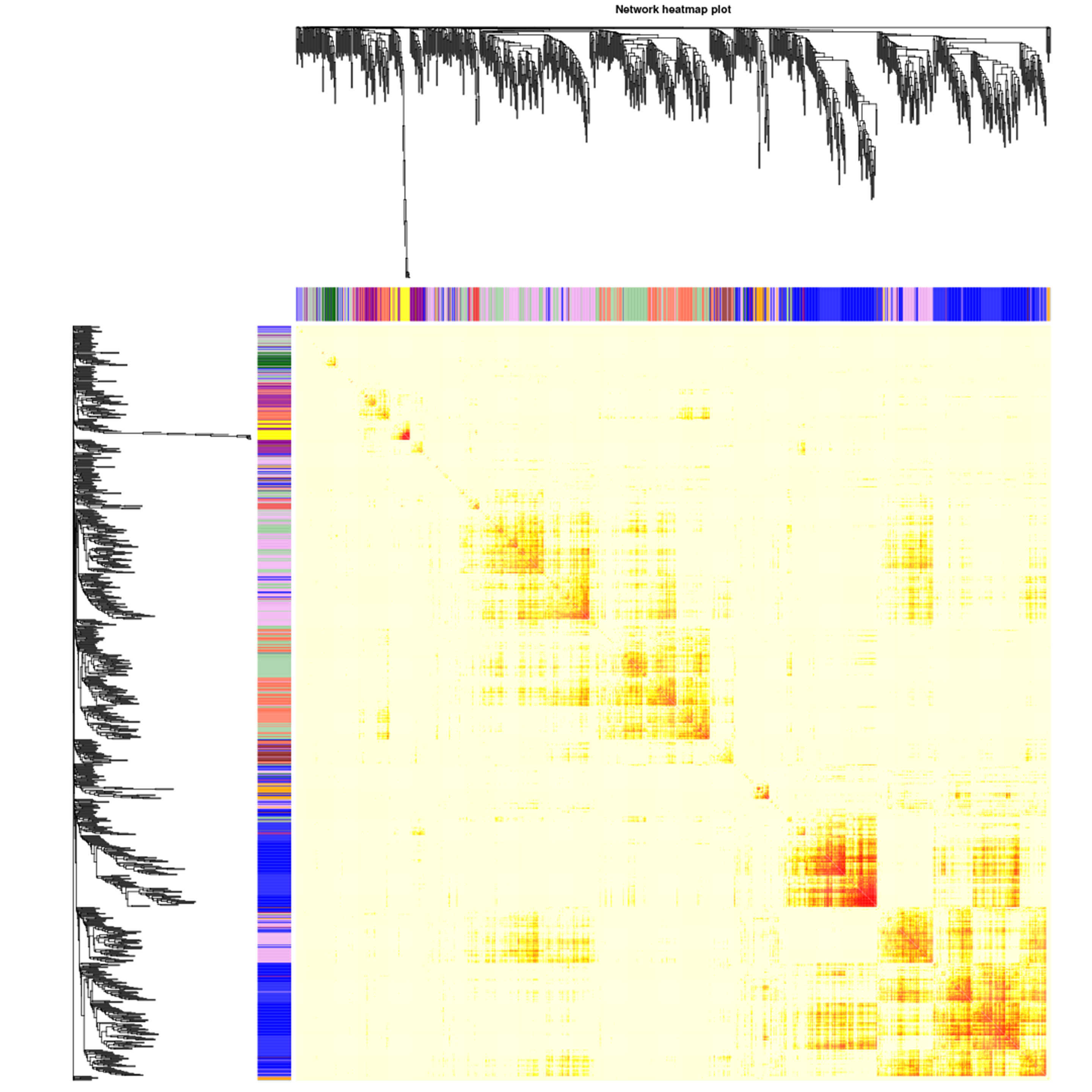

Supplement: Supplementary file 1 [file Image2.TIF]

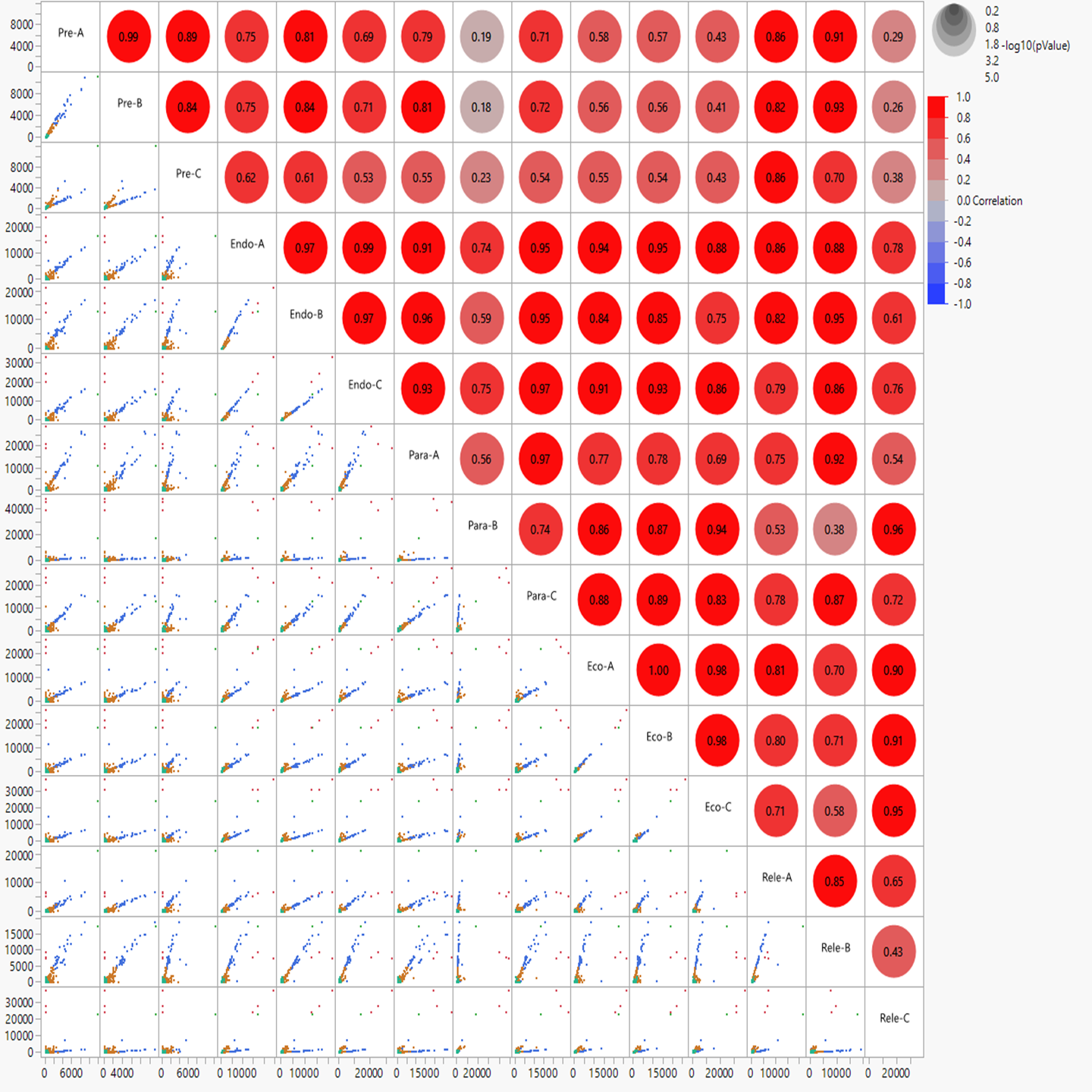

Supplement: Supplementary file 2 [file Image1.TIF]
